# Self-Reported Adverse Events of COVID-19 Vaccines in Polish Healthcare Workers and Medical Students. Cross-Sectional Study and Pooled Analysis of CoVaST Project Results in Central Europe

**DOI:** 10.3390/jcm10225338

**Published:** 2021-11-16

**Authors:** Arkadiusz Dziedzic, Abanoub Riad, Sameh Attia, Miloslav Klugar, Marta Tanasiewicz

**Affiliations:** 1 Department of Restorative Dentistry with Endodontics, Medical University of Silesia, 40-055 Katowice, Poland; martatanasiewicz@sum.edu.pl; 2 Czech National Centre for Evidence-Based Healthcare and Knowledge Translation, Institute of Biostatistics and Analyses, Faculty of Medicine, Masaryk University, Kamenice 5, 625 00 Brno, Czech Republic; abanoub.riad@med.muni.cz (A.R.); klugar@med.muni.cz (M.K.); 3 Department of Public Health, Faculty of Medicine, Masaryk University, Kamenice 5, 625 00 Brno, Czech Republic; 4 Department of Oral and Maxillofacial Surgery, Justus-Liebig-University, Klinikstrasse 33, 35392 Giessen, Germany; sameh.attia@dentist.med.uni-giessen.de

**Keywords:** COVID-19, adverse effects, BTN162 mRNA vaccine, vector vaccine, cross-sectional study, adverse reactions, Poland, healthcare workers, pooled analysis, prevalence

## Abstract

Background: Optimization of COVID-19 vaccination rate among healthcare personnel is of utmost priority to secure provision of uninterrupted care and to protect the most vulnerable patients. This study, as part of the global CoVaST project, aimed to assess the occurrence of short-term adverse events (SRAEs) of two most administered COVID-19 vaccines, mRNA-based (Pfizer-BioNTech and Moderna) and viral vector-based (AstraZeneca) in healthcare sector workers (HWs). Methods: A cross-sectional survey-based study was carried out for the first time among 317 Polish healthcare sector personnel and medical students using a validated and pre-tested questionnaire. The online questionnaire included 25 pre-tested, validated questions concerning demographic data, medical parameters, COVID-19-related anamneses, and local or systemic reactions (reactogenicity) associated with COVID-19 vaccination. Descriptive statistics, inferential tests and binary logistic regression were performed. Results: Out of the 247 participating HWs, 79.8% were females, and 77.5% received mRNA-based vaccines, while 24.5% received a viral vector-based vaccine. Cumulatively, 78.9% and 60.7% of the participants reported at least one local and one systemic SRAE respectively, following their COVID-19 first or second dose of vaccine. A wide array of SRAEs was observed, while pain at injection site (76.9%) was the most common local SRAE, and fatigue (46.2%), headache (37.7%), muscle pain (31.6%) were the most common systemic SRAEs. The vast proportion of local (35.2%) and systemic (44.8%) SRAEs subsided up to 1 day after inoculation with both types of vaccines. The mRNA-based vaccine versions seem to cause higher prevalence of local SRAEs, mainly pain within injection site (81.3% vs. 71.7%; *p* = 0.435), while the viral vector-based vaccine was linked with increased incidents of mild systemic side effects (76.7% vs. 55.3%; *p* = 0.004) after both doses. Pooled analysis revealed uniform results while comparing the prevalence of SRAEs in HWs as recipients in four central European countries (OR = 2.38; 95% CI = 2.03–2.79). Conclusions: The study confirmed the safety of commonly administered vaccines against COVID-19, which were associated with mild, self-resolving adverse events. No major vaccine-related incidents were reported which would affect every day functioning, significantly. The younger age group (below 29 y.o.) were associated with an increased risk of adverse events generally. The results enhanced current data regarding COVID-19 vaccination active surveillance in selected occupational groups.

## 1. Introduction

Public health experts, regulatory authorities, and advisory governmental bodies have been considering compulsory COVID-19 vaccination for eligible healthcare workers (HWs), including permanent and temporary personnel [1]. Maximization of the COVID-19 vaccination rate in HWs and, additionally, students attending Medical schools is deemed to be an evidence-based, rational process of public health agendas [2]. Healthcare workers have an exceptional duty of care towards patients, especially during a pandemic, that affects a vast majority of populations, causes serious health consequences, and death [3]. Equally, HWs and medical students have direct, frequent contact with vulnerable individuals who can be exposed to high risk of health problems. The awareness of the importance of vaccination programs in HWs groups influences their attitude towards public health preventive measures, reducing the risk of serious medical incidents. This seems particularly plausible when predicting a long-term anti-COVID-19 strategy involving subsequent doses of booster vaccines in the future [4].

By 30 October 2021, 3,008,294 COVID-19 cases with 76,875 deaths were reported in Poland, a country with a population amounting to 37 million [5]. The European Medicines Agency (EMA) conditionally approved the first anti-COVID-19 vaccine BNT162n2, known as the Pfizer-sBioNTech COVID-19 vaccine, on 21 December 2020 [6]. Until April 2021, four COVID-19 vaccines had been approved for use in Poland; Pfizer-BioNTech (BNT162b2, an mRNA-based vaccine, approved on 21 December 2020), Moderna (mRNA-1273, an mRNA-based vaccine, approved on 6 January 2021), AstraZeneca-Oxford (AZD1222, a non-replicating viral vector vaccine, approved on 29 January 2021, and Janssen (Ad26.COV.2.S, a non-replicating viral vector vaccine, approved 11 March 2021) [7,8,9,10]. In Poland, vaccines are authorised by the president of the Office for Registration of Medical Products, Medical Devices and Biocidal Products (URPL). On 30 October 2021, 39,058,861 vaccine doses were administered in Poland, leading to 19,962,570 people (51.27% of the total population) being fully vaccinated and 20,245,032 people (52.54%) receiving at least one dose of vaccine [11].

The National Vaccination Program in Poland, similar to other government programs in Europe, decided to prioritise HWs to receive the COVID-19 vaccine at the early stages of the national immunisation strategy, to enable uninterrupted care provision and to protect the most vulnerable groups of patients [12]. On 27 December 2020, the first cohort of HWs received the COVID-19 vaccine and the program rollout commenced in Poland as the main anti-COVID-19 strategy established by the Polish Ministry of Health (MoH). In Phase 0, frontline HWs, medical students, and critical public workers were prioritised for inoculation, using mRNA-based vaccines as they were only type available at the time.

While newly introduced vaccines are classified as novel pharmaceuticals, they are subjected to independent pharmacological-vigilance evaluation carried out by medical agencies, regulators, academic and clinical institutions. This process may involve passive, active or hybrid surveillance systems [13]. The active surveillance protocol comprises an approach similar to the Phase III approach and can be conducted in the form of surveys including vaccinated individuals and their self-reported adverse effects [14]. Until 17 September 2021, according to the National Epidemiological Inspectorate in Poland, 15,320 cases of post-vaccination adverse effects associated with COVID-19 vaccines were reported, in which 12,900 were of mild intensity, primarily erythema and short-lasting pain at the injection site [15].

The independent surveys dedicated to the active evaluation of post-vaccination side effects provide crucial data for infectious diseases surveillance. They support public health efforts to monitor anti COVID-19 vaccination programs and encourage specific groups in society to acquire adequate immunity [16]. Moreover, these surveys reduce apprehension towards any inoculation programs by providing a reliable evidence, based on benefits-risk evaluation to monitor adverse events and communicate safety information among healthcare providers, as well as regulators. Since mass-vaccination programs against COVID-19 commenced in December 2020, numerous survey-based studies have accomplished to investigate the prevalence and characteristics of the side effects associated with various, commonly used anti COVID-19 vaccines. These independent (active), Phase IV studies on mRNA-based and vector-based vaccines were conducted in 2021 on HWs groups in several regions, including Germany, the Czech Republic, Slovakia, Iraq, Italy, Malta, Jordan, Saudi Arabia, Poland, the UK, and the USA [17,18,19,20,21,22,23,24,25,26,27,28]. Their main findings, particularly regarding the intensity/duration of adverse effects, are generally in line with the results of the pivotal Phase III vaccine clinical trials carried out by the pharmaceutical companies and officially reported by the health regulators, such as the European Medicines Agency (EMA) in Europe and the Centers for Disease Control and Prevention (CDC) in the US [29]. However, these trials are designed to deliver crucial data about efficacy and may not detect some vaccine-related side effects in under-represented or excluded populations (e.g., medically compromised individuals). There is incoherence associated with the prevalence of the post-vaccination adverse events, likely to be related to a better health status of the volunteering recipients of the Phase III trials.

The healthcare sector, including the HWs, was the first to be involved in the Stage 0 COVID-19 vaccination programs in Poland, as they are most at risk of being infected with COVID-19. Moreover, the non-vaccinated healthcare personnel may be of potential risk for exposed vulnerable patients. Such personnel are considered to be well acknowledged and aware of medical problems and are able to recognise and distinguish clinical symptoms. According to the information provided by the main Polish professional chambers and councils, in the middle of 2021 the total number of fully registered medical doctors was 181,002, the number of dentists was 37,000, and the number of pharmacists 33,988.

The recent cross-sectional study conducted in 2021 in Poland and Germany [30,31] revealed that respectively 51.9%/59.5% of the surveyed respondents, including HWs were willing to become vaccinated, 17.4%/21.4% were hesitant but might consider it in future, and 24.4%18.7% were against any inoculation. This indicates the need for the constant reinforcement of new strategies to reduce vaccine hesitancy, even in staff in the healthcare sector. Szmyd et al., (2021) observed that the vast majority of HWs in Poland declared their willingness to be vaccinated, compared to the control group (82.95% vs. 54.31%). However, this level of willingness remains unsatisfactory [32]. Fear related to unexpected adverse reactions to the anti-COVID-19 vaccine was commonly reported [31,32,33,34,35] and is one of the crucial factors of COVID-19 vaccine hesitancy that needs to be addressed and verified by well-designed studies. Hence, online surveys rendering various aspects of SRAEs provide reliable, transparent evidence regarding the safety of vaccines and their efficiency at population and individual level, enhancing the recipients’ confidence and equally, reducing vaccine hesitancy.

It is expected that the long-term effect of mass vaccination programs and associated reports regarding their safety profile obtained from independent studies, will also have a positive impact on healthcare aspects indirectly affected by COVID-19 pandemic, such as diagnostics and treatment of various medical conditions.

The primary aim of the cross-sectional survey carried out on Polish HWs and medical students was to provide an in-depth assessment on the prevalence of the reactogenicity of the COVID-19 vaccines, as well as their characteristics, including gender/age predilection, clinical manifestations, duration and demographic interrelations between variables. The analysis of the data allowed independent, reliable surveillance of post-vaccine adverse reactions among HWs in Poland as a part of the CoVaST multi-centre consortium. The obtained information was compared with results from the CoVaST projects in European countries exhibiting a similar profile of socio-demographic and healthcare systems that used the same protocol/study agenda.

## 2. Materials and Methods

### 2.1. Design

This post-marketing trial had been designed as a cross-sectional survey-based study that aimed to evaluate the short-term SRAEs of COVID-19 vaccines among Polish HWs. The study utilized a validated questionnaire that was designed and circulated online through SurveyMonkey (Momentive Inc, San Mateo, CA, USA 2021) [36]. The target population was recruited using a snow-balling technique (non-random sampling) and the study protocol was registered a priori at the United States (US) National Library of Medicine (NLM) under the identifier NCT04834869: COVID-19 Vaccines Safety Tracking (CoVaST) [37].

### 2.2. Participants

The target population of this study was the HWs in the Republic of Poland who received COVID-19 vaccines among the priority groups during the first quarter of 2021 [38]. A uniform resource locator (URL) and a quick response (QR) code were sent to potential respondents as they were able to download it from CoVaST project promoting sources, such as Medical Universities websites, scientific societies and professional regulatory bodies. The recruitment process took place during April and May, 2021, and it was subsequently extended to June 2021. The response rate was 78% defined as number of properly filled online forms/number of webform access.

Participation in this study was voluntary; therefore, the participants received no financial compensation or any other means of incentives in order to minimize both selection and information biases. The participants were able to withdraw from the study anytime without the need to justify their decision. The participants who did not complete the questionnaire properly were excluded from the final analyses.

### 2.3. Instrument

The self-administered questionnaire (SAQ) used in this study was adapted and validated from previous studies on side effects of COVID-19 vaccines and the safety reports of COVID-19 vaccines manufacturers that were published by the US Centers for Disease Control and Prevention (CDC, Atlanta, GA, USA) and the European Medicines Agency (EMA, Amsterdam, The Netherlands) [39,40,41,42,43,44].

The SAQ consisted of 22 multiple-choice items classified into four categories; (a) demographic data including gender, age, profession, and jurisdictional region; (b) medical anamnesis data including chronic illnesses and medical treatment taken regularly; (c) COVID-19-related anamnesis data including previous infections, type of vaccine, and number of doses; (d) local, systemic, oral, and skin-related SRAEs of COVID-19 vaccines and the palliative drugs used after vaccination.

The validation and reliability testing processes of the SAQ were described in detail previously [39]. The psychometric properties of the SAQ were satisfactorily high; therefore, dual forward translation and a review panel of experts were only needed to generate an equivalent Polish version of the SAQ, which demonstrated a substantial level of reliability with mean Cohen’s kappa coefficient of 0.89 ± 0.13 (0.54–1) [39,45].

### 2.4. Ethics

The study protocol was thoroughly reviewed and approved by two ethical committees: the Ethical Committee of the Medical University of Silesia (Katowice, Poland, Ref. PCN/CBN/0022/KB/161/21) and the Ethical Committee of the Faculty of Medicine at Masaryk University (Brno, Czech Republic) on 20th January 2021 (Ref 2/2021). Additionally, the study was carried out in accordance with the Declaration of Helsinki for scientific research involving human subjects, and it was reported according to the Strengthening the Reporting of Observational Studies in Epidemiology (STROBE) guidelines for cross-sectional studies [46,47].

All the participants had to give their informed consent digitally at the beginning of the questionnaire, and those participants who did not provide their consent were automatically disqualified from the study. Data collection and processing were carried out in accordance with the European Union (EU) General Data Protection Regulation (GDPR); therefore, no identifying personal data were collected from the participants [48].

### 2.5. Analysis

The Statistical Package for the Social Sciences (SPSS) version 27.0 (SPSS Inc. Chicago, IL, USA, 2020) was used in all statistical tests that were executed in this study. At the beginning, the normality of the data was examined using Shapiro–Wilk test with a significance level of (*p*) < 0.05.

Descriptive statistics was conducted for presenting the demographic variables (gender, age, profession, and region), anamnestic variables (chronic illnesses, medical treatments, COVID-19 infection, vaccine type, and vaccine doses), and SRAEs prevalence, onset and duration, using frequencies (*n*), percentages (%), and central tendency measures e.g., mean (*μ*) and standard deviation (*SD*).

Consequently, inferential tests were executed to evaluate the association between SRAEs prevalence, onset, and duration and demographic variables (age and gender) using chi-squared test (*χ*^2^) and Fisher’s exact test for cases with expected frequency below 5. All the comparisons were carried out on the basis of vaccine type: mRNA-based vaccines versus viral vector-based vaccines.

Finally, binary logistic regression for the incidence of local and systemic SRAEs was used to evaluate the potential demographic and medical predictors of adverse effects of administering COVID-19 vaccines. All the inferential tests were run with the assumption for a confidence interval (*CI*) of 95% and significance level of (*p*) < 0.05.

### 2.6. Pooled Analysis of Results of CoVaST Project Studies Conducted in Central Europe

The synthesis of data concerning the prevalence of local vs. systemic SRAEs occurring after 2nd dose of mRNA vaccine, depending on gender, reported by CoVaST surveys in Czech Republic, Slovakia, Germany, and Poland was carried out using weighted pooled analysis and RevMan 9.0v software, applying fixed effect statistics (odds ratio—OR, and risk ratio—RR).

## 3. Results

### 3.1. Demographic Characteristics

A total of 315 respondents accessed the digital questionnaire and provided their consent to participate, while 68 of them were excluded for various reasons; 8 were not vaccinated, 39 withdrew before answering any question, and 21 did not answer the required questions concerning COVID-19 SRAEs.

Therefore, only 247 participants were included in the final analyses and they were divided into two main groups; recipients of mRNA-based vaccine recipients (179 recipients of BNT162b2, commonly known as Pfizer-BioNTech COVID-19 vaccine, and 8 recipients of mRNA-1273 commonly known as Moderna COVID-19 vaccine) and recipients of viral vector-based vaccine recipients (58 recipients of ChAdOx1 nCoV-19, commonly known as AstraZeneca-Oxford COVID-19 vaccine, and 2 recipients of Ad26.COV2.S, commonly known as Janssen COVID-19 vaccine).

The vast majority (79.8%) of the participants were females, and none of them refused to disclose their gender. The mean age was 29.83 ± 11.72 (19–71) years of age. Medical students represented majority of the participants (47.8%), followed by dentists (13.8%) and physicians (12.6%). The most represented region was Śląskie (Silesian Province), followed by Małopolskie (Lesser Poland Province), Dolnośląskie (Lower Silesian Province), and Swiętokrzystkie (Holy Cross Province) (Table 1).

### 3.2. Medical Anamnesis

About one quarter of the participants (25.9%) reported having at least one chronic illness, and there was no significant difference (*χ*^2^ = 0.274; *p* = 0.600) between the total number of recipients of mRNA-based vaccine (26.7%) and the recipients of viral vector-based vaccine (23.3%). However, there was statistically significant difference in reported hypertension cases (*p* = 0.025), including 7.5% recipients of mRNA-based vaccine vs. no recipients of vector-based vaccine. Thyroid-related diseases (13%) were the most common illnesses among the participants, followed by chronic hypertension (5.7%), psychological distress (4.5%), and chronic obstructive pulmonary disease (2.4%).

Over two-fifths of the participants (42.9%) reported taking medications regularly, and there was no significant difference (*χ*^2^ = 0.141; *p* = 0.708) in total between the recipients of mRNA-based vaccine (42.2%) and the recipients of viral vector-based vaccine (45%). Coherently, to medical history results, there was a statistically significant difference (*p* = 0.043) in the numbers of persons being on antihypertensive medications (6.4% recipients of mRNA-based vaccine vs. no recipients of vector-based vaccine). Thyroid hormone supplements (21.6%) were the most used type of medication, followed by contraceptives (17.4%), antihistamines (6.5%), antidepressants (5.7%), and antihypertensives (4.9%) (Table 2).

### 3.3. COVID-19-Related Anamnesis

Overall, 77.3% of the participants received two doses of COVID-19 vaccine. While 75% of recipients of viral vector-based vaccine received only one dose, 5.9% of the mRNA-based vaccine group received only one dose (*χ*^2^ = 123.779; *p* < 0.001). Six participants reported being infected with COVID-19, five before receiving the first dose, and only one participant was infected after receiving the first dose of Pfizer-BioNTech vaccine (Table 3).

### 3.4. Local Side Effects

A total of 195 (78.9%) participants reported at least one local adverse event, related to the injection site, and the prevalence among the mRNA-based vaccine group (81.3%) was higher than in the viral vector-based vaccine group (71.7%) but not statistically significant (*χ*^2^ = 2.528; *p =* 0.112). Pain at injection site was the most common local SRAE (76.9%). While 42% of local SRAEs occurred exclusively after the first dose, only 5.2% occurred exclusively after the second dose.

The vast majority (82.7%) of the local SRAEs resolved within 1–3 days after receiving the vaccine. The local SRAEs tended to be of longer duration among the viral vector-based vaccine group, as 16.7% of them had their local SRAEs resolved within the first day compared to 40.4% of the mRNA-based vaccine recipients (*χ*^2^ = 9.996; *p* = 0.002). Moreover, 16.7% of the viral vector-based vaccine group had their local SRAEs resolved within one week, compared to only 1.3% of the mRNA-based vaccine group (*p* = 0.001; 2-S Fisher’s exact test) (Table 4).

### 3.5. Systemic SRAEs

A total of 150 (60.7%) recipients reported at least one systemic SRAE, and the prevalence among the mRNA-based vaccine group (55.6%) was significantly (*χ*^2^ = 8.441; *p* = 0.004) lower than in the viral vector-based vaccine group (76.7%). Fatigue was the most common systemic SRAE (46.2%), followed by headache (37.7%), muscle pain (31.6%), chills (31.2%), fever (28.7%), and joint pain (21.9%).

**Figure 1:**
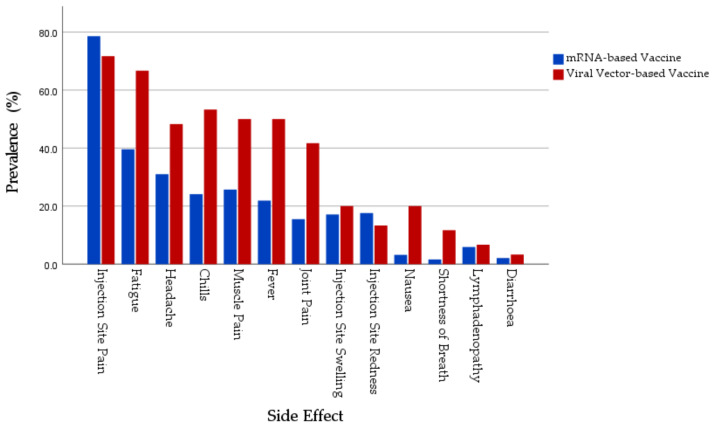
Prevalence of COVID-19 vaccines SRAEs reported by Polish healthcare workers.

In all the solicited systemic SRAEs, the recipients of viral vector-based vaccine recipients were more affected, in comparison to the recipients of mRNA-based vaccine recipients: fatigue (66.7% vs. 39.6%), headache (58.3% vs. 31%), muscle pain (50% vs. 25.7%), joint pain (41.7% vs. 15.5%), fever (50% vs. 21.9%), chills (53.3% vs. 24.1%), nausea (20% vs. 3.2%), diarrhea (3.3% vs. 2.1%), lymphadenopathy (6.7% vs. 5.9%), and shortness of breath (11.7% vs. 1.6%). Although, anaphylaxis was solicited in the SAQ, no participant reported experiencing it (Figure 1).

Among the mRNA-based vaccine recipients, 45.1% reported systemic adverse events exclusively after the second dose and 39.2% reported them after both doses. A vast majority (84.8%) of systemic SRAEs resolved within 1–3 days after receiving the vaccine. There was no significant difference between the mRNA-based and the viral vector-based vaccine groups in terms of systemic SRAEs duration (Table 5).

### 3.6. Orofacial and Skin-Related SRAEs

Orofacial and skin-related side effects were reported by 7.3% of the participants with a significant difference (*p* = 0.048; 2-S Fisher’s exact test) between the group receiving mRNA-based vaccine (5.3%) and the viral vector-based vaccine group (13.3%).

Dysgeusia/taste disturbance was the most common orofacial SRAE (2.4%) while skin rash was the most common skin-related side effect (2.4%). The participants described their taste disturbances as “metallic taste, change of taste, and bitter taste”.

Xerostomia (dry mouth) was not solicited in the SAQ; even though, two participants reported having it after being injected with COVID-19 vaccines. Although facial nerve palsy was solicited in the SAQ, no participant reported having it after receiving COVID-19 vaccines (Table 6).

### 3.7. Over the Counter, Alleviating Drugs Taken after Vaccination

When asked about the analgesic/anti-inflammatory drugs, less than two-fifths of the participants (38.5%) reported taking medications to control the intensity of post-vaccination side effects. The recipients of viral vector-based vaccine (56.7%) had significantly (*χ*^2^ = 11.079; *p* = 0.001) higher level of palliative drugs consumption than the recipients of mRNA-based vaccine recipients (32.6%). The most commonly used drug was paracetamol (25.5%), followed by Ibuprofen (10.1%) and Pyralgin (3.6%) (Table 7).

### 3.8. COVID-19 Vaccines SRAEss by Gender

**Figure 2:**
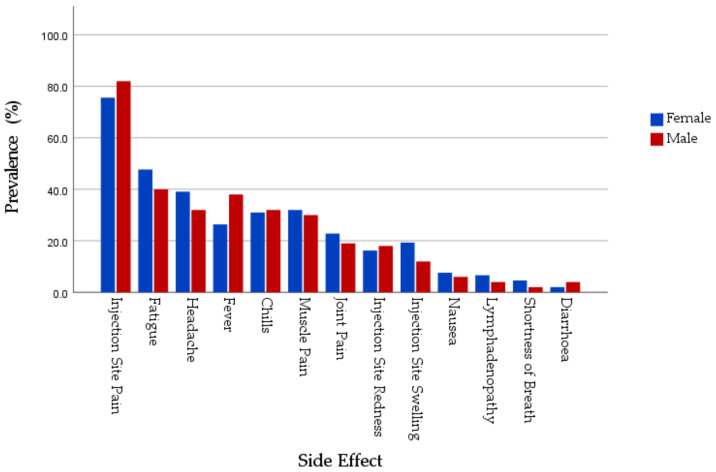
Gender-specific distribution of COVID-19 vaccines adverse events.

When comparing the prevalence of SRAEs in total by gender, no significant difference was found as regards local SRAEs (*χ*^2^ = 0.351; *Sig.* = 0.553) or systemic SRAEs (*χ*^2^ = 0.014; *p* = 0.906) (Figure 2).

In the group injected with mRNA-based vaccine group, females had higher prevalence of swelling at injection site (20.3% vs. 5.1%, statistically significant with *p* = 0.026), redness at injection site (18.2% vs. 15.4%), fatigue (40.5% vs. 35.9%), headache (32.4% vs. 25.6%), and muscle pain (26.4% vs. 23.1%). Overall, males, mRNA recipients had slightly higher prevalence of local side effects (84.6% vs. 80.4%, *p* = 0.549) and systemic SRAEs (61.5% vs. 54.1%, *p* = 0.403).

Among the group that received viral vector-based vaccine group, females had higher prevalence of fatigue (69.4% vs. 54.5%), headache (59.2% vs. 54.5%), joint pain (44.9% vs. 27.3%), chills (55.1% vs. 45.5%), nausea (22.4% vs. 9.1%), and lymphadenopathy (8.2% vs. 0%). Overall, in this group, females (81.6%) were more frequently affected by the systemic SRAEs compared to their male counterparts (54.5%) counterparts; however, the difference was not statistically significant (*p* = 0.107; 2-S Fisher’s exact test).

Females had a higher level of palliative drugs consumption (40.6% vs. 30%) compared to males, either the group receiving mRNA-based vaccine group (34.5% vs. 25.6%) or the group exposed to viral vector-based vaccine (59.2% vs. 45.5%). This discrepancy was not statistically significant (Table 8).

### 3.9. COVID-19 Vaccines SRAEs by Age

**Figure 3:**
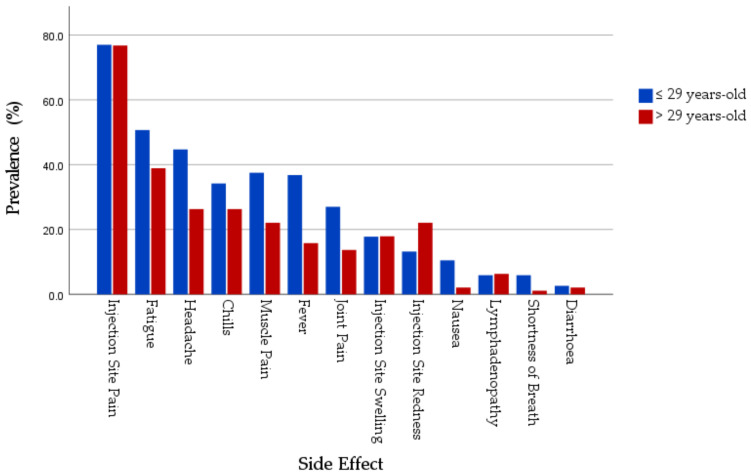
Distribution of COVID-19 Vaccines SRAEs by age.

When comparing the side effects prevalence by age, no significant difference was found in local SRAEs (*χ*^2^ = 0; *p* = 1.000) between the young age group (≤29 years-old) and the old age group (>29 years-old). As regards systemic adverse events, the young age group (65.8%) had a significantly (*χ*^2^ = 4.244; *p* = 0.039) higher prevalence compared to the old age group (52.6%) (Figure 3).

In the group receiving mRNA-based vaccine group, the young age group had higher prevalence of pain at injection site (80% vs. 77%), swelling at injection site (18% vs. 16.1%), fatigue (41% vs. 37.9%), headache (37% vs. 24.1%), muscle pain (29% vs. 21.8%), joint pain (17% vs. 13.8%), and fever (27% vs. 16.1%). Overall, the young age group had higher prevalence of local SRAEs (83% vs. 79.3%) and systemic SRAEs (59% vs. 51.7%).

Among the viral vector-based vaccine recipients, the young age group had higher prevalence of fatigue (69.2% vs. 50%), headache (59.6% vs. 50%), muscle pain (53.8% vs. 25%), joint pain (46.2% vs. 12.5%), fever (55.8% vs. 12.5%), chills (53.8% vs. 50%), nausea (23.1% vs. 0%), lymphadenopathy (7.7% vs. 0%), and shortness of breath (13.5% vs. 0%). Overall, the young age group (65.8%) was more profoundly affected by systemic SRAEs, as compared to the old age group (52.6%); the difference was statistically significant (*p* = 0.039; 2-S Fisher’s exact test).

The young age group had a non-significantly higher level of palliative drugs consumption (42.1% vs. 32.6%) compared to the old age group (Table 9).

### 3.10. Predictors of SRAEs of COVID-19 Vaccines

The binary logistic regression method revealed that females had odds ratio (OR) of 3.704 (CI 95%: 0.923–14.866) in experiencing systemic adverse events following viral vector-based vaccine, higher in comparison with their male counterparts. Moreover, females had a lower OR of experiencing local adverse events following mRNA-based vaccines (OR = 0.746; CI 95%: 0.286–1.948) and viral vector-based vaccines (OR = 0.938; CI 95%: 0.217–4.055).

The young age group was associated with increased likelihood of systemic SRAEs (OR = 1.731; CI 95%: 1.025–2.923) after mRNA-based vaccines (OR = 1.343; CI 95%: 0.752–2.397) and viral vector-based vaccines (OR = 2.236; CI 95%: 0.461–10.841).

Chronic illness increased the risk of experiencing SRAEs after COVID-19 vaccination, especially after viral vector-based vaccines; local SRAEs (OR = 2.903; CI 95%: 0.575–14.654) and systemic SRAEs (OR = 2.118; CI 95%: 0.413–10.865). Similarly, taking regular medications increased the risk of experiencing side effects after COVID-19 vaccination, especially after the viral vector-based vaccine; local adverse events (OR = 3.737; CI 95%: 1.049–13.319) and systemic adverse events (OR = 1.650; CI 95%: 0.479–5.684).

Experiencing systemic adverse events increased greatly the risk of palliative drugs consumption in both vaccines recipients (OR = 22.128; CI 95%: 9.105–53.779), while local adverse events had a less impact on palliative drugs consumption (lesser extent impact) (OR = 5.287; CI 95%: 2.272–12.306) (Table 10).

### 3.11. Pooled Analysis Results from CoVAST Studies Conducted in Czech Republic, Slovakia, Germany, and Poland

**Figure 4:**
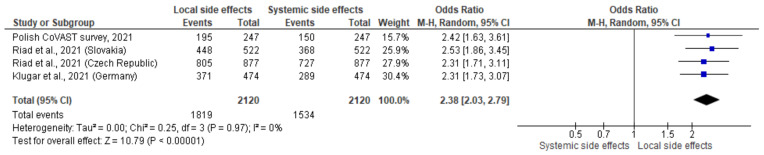
Forest plot of pooled analyses of total local and systemic post-vaccination adverse events in four European countries, participants of CoVAST project.

**Figure 5:**
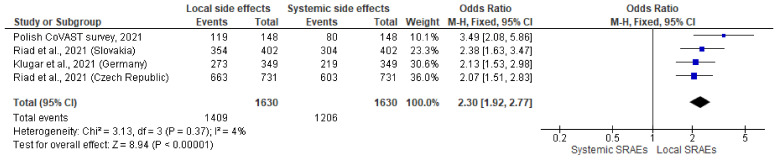
Forest plot of gender-specific pooled analysis of local and systemic adverse effects (mRNA vaccine) in four European countries (females only).

Relatively uniform results were observed when comparing the prevalence of total SRAEs in HWs recipients in four Central European countries which contributed to CoVAST project. Local SRAEs were significantly more dominant in all analysed studies, with favourable OR = 2.38 (95% CI = 2.03–2.79) (Figure 4). Minimal level of heterogenicity was found when assessing the fractions of local vs. systemic SRAEs in female HWs in Czech Republic, Slovakia, Poland, and Germany (I^2^ = 4%, *p* = 0.37, Figure 5). The prevalence of SRAEs observed in Polish HWs was also higher in relation to CoVAST studies (OR = 3.49; CI 95%: 2.08–5.86).

## 4. Discussion

This survey-based study, which was part of the CoVaST surveillance project, was designed to evaluate and compare the safety and reactogenicity of anti-COVID-19 vaccines, when providing detailed characteristics of post-vaccination self-reported side effects in the HWs in Poland. SRAEs associated with two anti-COVID-19 vaccines were compared; the mRNA-based vaccine (Pfizer-BioNTech) and the viral vector-based vaccine (AstraZeneca), revealing moderate differences in the prevalence of local and systemic SRAEs in gender and age groups of HWs, following the first, second, and both doses of immunisation. This was one of the first comprehensive surveys that included HWs in Poland, as well as a cohort of Polish students attending medical universities in evaluating the SRAEs of two COVID-19 vaccines. Within the limitations of our survey, the results obtained suggest that age, gender, and type of vaccine are the main predictors and confounders affecting the prevalence and severity of SRAEs. Only eight participants of our survey (2.5%) declared unvaccinated status.

The results of our study are homogenous with those of other cross-sectional survey-based studies carried out in Central Europe by the CoVaST project participants in Germany [28], the Czech Republic [17], and Slovakia [19], which used standardised and validated protocol [39]. It needs to be stressed that the average structure of healthcare systems in the above-mentioned countries seems comparable, consisting mainly of the national public healthcare sector. While independent studies showed the reasonable safety of the current programs of inoculation against COVID-19 using representative samples, they are expected to impact the global vaccination rate in a positive way (Table 11).

Considering the study population, a substantial proportion of the total 78.6% of Polish HWs and medical students declared at least one local adverse event after being vaccinated with both doses of either the mRNA-based vaccine (BNT162b2)—81.3% or the vector-based vaccine—71.7% of respondents (*p* = 0.012). These SRAEs were unremarkable, minor, short-lasting (1–3 days maximum), and self-resolving, while pain at the injection administration site was the most common local SRAE (78.6% vs. 71.7%, respectively), followed by swelling at the injection site, and redness at the injection site. Almost half (47.7%) of local SRAEs lasted up to three days, and 35.2% self-resolved within the first day after the vaccination. Equally, around half of systemic SRAEs, as a result of either the mRNA or the vector-based vaccine, lasted for 24 h (44.8% in total) or resolved within three days (40%). Among participants, in total, 60.7% of all Polish HWs reported at least one post-vaccination systemic adverse event, with a substantial difference in prevalence between the mRNA vaccine (55.6%) and vector-based ones (76.7%). Fatigue (46.2%), headache (37.7%), and muscle pain (31.6%) were the most frequent SRAEs. These findings demonstrated that anti-COVID-19 vaccines approved in Europe possess a generally safe profile, causing mainly limited, and self-resolving systemic adverse reactions. Mathioudakis et al., (2021) confirmed that the mRNA vaccine was associated with mostly local symptoms and a lower prevalence of systemic side effects (RR < 0.6) [49].

The previous study conducted in Poland among HWs at the beginning of 2021 showed that in 78% (1253 in total) of recipients of the first dose of the anti-COVID-19 vaccine, the most prevalent side effect was, similarly to our results, pain at the site of injection and pain in the arm [18]. About 50% of recipients experienced a wide range of local and systemic symptoms after the second dose, including general fatigue (30%), swelling at the injection site (24.5%), malaise (21.3%), redness at the injection site, headache, muscle and joint pain, fever and chills. Jezkowiak et al. [18] observed that episodes of COVID-19 contraction in the past influenced the occurrence of side effects in a negative way, with more severe adverse reactions after the first dose.

A dominant female gender profile (79.8%) proved the national representativeness of the sample, as the vast majority of HWs in Poland are women and showed an unequal distribution of gender. Gender discrepancy was more apparent in Polish HWs compared to results obtained from other CoVaST studies ([17,18,19,28], Figure 5) and independent, non-commercial surveys [20,21,22,23,24,25,26,27,28,50]. Gender-related discrepancies of the prevalence of post-vaccination SRAEs revealed a higher likelihood of female HWs experiencing and reporting systemic SRAEs associated with the vector-based vaccine (OR = 3.704, CI 95% 0.92–14.86), although the differences were not statistically significant. This observation is coherent with previous results of the Phase IV trials of COVID-19 vaccines from the UK and Saudi Arabia [20,51]. Interestingly, it has been reported that female HWs in Italy were attributed with a more potent immune response to the vaccine and serological parameters following the COVID-19 vaccination, suggesting a correlation with more frequent post-vaccination SRAEs [52].

Young age (<29 years old) was a risk factor and a predictor contributing to an increased incidence rate of systemic SRAEs, revealed by high adjusted odds of SRAEs (OR = 1.731; CI 95%: 1.025–2.923). On the contrary, no significant difference was found in the prevalence of local SRAEs between two age groups. These findings resemble the results of other studies, demonstrating that systemic SRAEs were more frequent among the groups of vaccine recipients aged ≤55 years of BNT162b2 in the United Kingdom [20]. Moreover, our data are in line with Cuschieri et al., report which demonstrated that the post-BNT162b2 vaccine SRAEs in Maltese HWs were intensified in persons aged under 45 years, regardless of the gender influence [26]. In addition, a study conducted in Iraq concluded that middle-aged HWs were more susceptible to vaccination-related SRAEs [23].

Both local and systemic post-vaccination SRAEs (BNT162b2) were also considerably more prevalent among HWs aged <43 years in a recent study conducted in the Czech Republic [17]. The age-related discrepancies in SRAEs following COVID-19 vaccination were found in the clinical trials conducted by the main manufacturers of the vaccines (Phase III) and reported by the Centers for Disease Control and Prevention. Recipients of the BNT162b2 vaccine aged under 55 years had more frequent local and systemic adverse events [29]. Cumulatively, the occurrence of adverse effects after the first dose of the COVID-19 vaccine only, in the young group (<29 years old) was apparent, compared to the older cohort (*p* < 0.001).

As expected, the reported use of symptom-relieving medications was a high predictor of systemic SRAEs for both the mRNA and the AZD1222 vector-based vaccines (OR = 18.22 and 33.00 respectively, 22.128 in total, CI 95% 9.10–53.77). Existing, chronic health conditions and prolonged pharmacotherapy were correlated with a more frequent occurrence of both local and systemic SRAEs associated with vector-based vaccines, however these differences were not statistically significant. Similar findings were reported by Mathioudakis et al., (2021), who comprehended that participant diagnosed with COVID-19 in the past and now recovered had a higher risk of both local and systemic post-vaccination incidents, including severe events leading to hospitalisation [49]. The factual link between chronic diseases and the incidence post-vaccination side effects has not yet been established yet. The recipients of COVID-19 vaccines who reported that they were diagnosed with coexisting medical conditions appeared to have lower adjusted odds of local SRAEs compared to the adjusted odds of systemic ones (1.391 vs. 1.106, respectively). It has been hypothesised, that adverse reactions following vaccination could be affected by underlying or undiagnosed health problems [53].

On the contrary, an impaired immune response due to coexisting illnesses can be associated with lessened, attenuated immune reactivity leading to reduced adverse events [54]. Similarly, there is a lack of sufficient data regarding the interactions of COVID-19 vaccines and common pharmacological agents, such as contraceptives, antihypertensive drugs, or antihistamine medications. Current medications taken by participants did not seem to be predictors of either local, or systemic SRAEs.

To date, scientific reports indicate the differences in the reactogenicity caused by two main globally available COVID-19 vaccines, based on the following technologies: (i) the mRNA-based one, (ii) the non-replicating viral vector one. The modern, state-of-the-art mRNA-based technology uses mRNA molecular elements to provide genetic information to induce the production of antibodies against the spike protein antigen. The BNT162n2 vaccine uses mRNA, nucleoside-modified messenger RNA (modRNA), encoding the viral spike (S) glycoprotein of SARS-CoV-2, which constitutes the active ingredient. On the contrary, the vector-based vaccine delivers the virus antigen itself in order to initiate the expected immune response and the production of SARS-CoV-2 antibodies. AZD1222 is described as a recombinant, replication-deficient chimpanzee adenovirus vector encoding the SARS CoV 2 spike glycoprotein, produced in genetically modified human embryonic kidney (HEK) 293 cells [55,56]. Due to the rapid development of those vaccines in 2020 and their conditional approval, they were the subject of anti-vaccine campaigns and vaccination hesitancy among populations. Official clinical trials showed that these two most predominantly used COVID-19 vaccines have acceptable safety profiles [57,58].

The differences in prevalence of SRAEs, depending on the type of vaccine, were also confirmed in the CoVaST project conducted in Germany [28]. In this project, compared to our results, 61% of recipients declared a wide range of systemic SRAEs after the mRNA vaccine vs. 55.6% of Polish HWs and 87.2% after the viral vector vaccine vs. 76.7% of Polish HWs (*p* < 0.001). The overall profile of vaccine anamnesis in German HWs, as a result of the sequence of the vaccination program in European countries, including Poland, reflected the prioritisation of front-line workers when only the mRNA vaccine was available and approved at the beginning of 2021. Accordingly, 90.3% of HWs in Germany and 94.1% of HWs in Poland received two doses of the mRNA vaccine. In total, 77.3% of Polish HWs respondents were fully vaccinated with either the mRNA or the vector-based vaccines.

According to Zdziarski et al. [59], Polish medical professionals (‘doctors’) declare an enthusiastic, pro-vaccination attitude after receiving their anti-COVID-19 vaccines. The vast majority of respondents, from 52.1% to 62.5% depending on their age group, stated that they did not feel worried about the side effects of vaccine received. In another study carried out among HWs in Poland, 76.2% respondents indicated that vaccination against COVID-19 is strongly needed. Moreover, 74% would like to receive a booster dose of the vaccine against COVID-19 [18]. This positive approach of Polish HWs seems consistent with the attitude of medical professionals in the UK who were willing to be vaccinated (71.7%) and demonstrated pro-vaccine behaviours [60], and this was also seen in other countries [30,31,32,33].

Even though the results of the survey performed by Babicki et al., (2021) demonstrated that people with a higher level of education and healthcare workers have a more favourable attitude toward vaccination against COVID-19 [30], there is still a growing concern related to the unvaccinated healthcare personnel. Regardless of the fact that more evidence emerged showing the safety of the COVID-19 vaccine, a certain percentage of the population still exhibit fear of post-vaccination side effects [33,34]. Vaccine hesitancy was especially apparent during the initial stage of the vaccination programs [61]. This attitude is maintained despite the evidence-based data confirming the safety and effectiveness of anti-COVID-19 vaccines. According to Bergwerk at al., (2021), the incidents of most mild/asymptomatic COVID-19 infections in fully vaccinated US healthcare workers is correlated with neutralising antibodies levels [62]. A recent systematic review evaluating the HWs attitudes towards COVID-19 vaccination showed a significant variation of vaccine acceptance ranging from 27.7% to 77.3% [63]. Di Gennaro et al., (2021) concluded that sufficient investment in HWs vaccine-related education is urgently needed, as 26% of HWs respondents in Italy were not convinced to receive COVID-19 vaccination [64]. The main reason of vaccine hesitancy was lack of the trust in safety profile of vaccine.

Vaccine hesitancy is deemed particularly apparent in young populations, including students [65,66]. Adams et al., (2021) stated that due to a reasonably high level (24%) of young adult reluctant to receive vaccine against COVID-19, targeted promotion of vaccination addressing safety concerns (56% respondents) is well justified and desired [67]. Hence, special efforts must be taken by universities to promote and enhance the willingness of young persons to be vaccinated. The students of medical, dental, pharmaceutical, and physiotherapy faculties, in particular, must be encouraged to be fully vaccinated before clinical sessions involving direct contacts with patients, particularly with immune-compromised individuals. This aspect has been raised in a global study involving 6639 dental students from 22 countries, which found that the COVID-19 vaccine acceptance rate was unsatisfactory, with 22.5% of respondents hesitant, and 13.9% of those having declined vaccines [68].

### 4.1. Strengths and Limitations

The selection of HWs as a target population allowed for accuracy of critical data reporting, as this group, by default, possesses adequate levels of health literacy, as well as scientific interest [69]. The vast proportion of young respondents participating in the study is deemed unique compared to similar surveys. The standardised, consistent survey methodology, as provided by the CoVaST project in all participating countries, guarantees the reliability of data acquisition and comparison between targeted populations. The consistent results obtained in four European countries, as revealed by pooled analysis, validated the reliability and usefulness of standardised methodology of the CoVaST multi-national project.

The primary limitation of this survey is related to the self-reported data and information about COVID-19 vaccine side effects, provided instead of the objective information reported by healthcare professionals following formalised, unbiased assessment. The moderate sample size of the surveyed group may have an impact on the overall data, results analysis, and representativeness. Due to the primary aim of this study, the selected sample and its demographic characteristics were not homogeneously distributed or comparable with the population profile. The predominance of the mRNA-based BNT162b2 COVID-19 vaccine was the result of the Phase I distribution of this vaccine among the HWs at the beginning of 2021, a decision made by decision-makers and public health officials. As this study applied an online survey only, the inclusion of HWs with limited internet access might be compromised. The uncompleted submission of questionnaire questions resulted in considerable proportion of participants who had to be excluded. The unexpected hesitancy in study participation was reflected by a substantial proportion of uncompleted surveys, which revealed the attitude of some respondents towards independent data reporting.

It was expected that HWs were more likely to be cautious about health matters, as compared to the general population. Hence, the external validity might verify the data obtained. An additional limitation is related to the complex layout of the survey instrument applied, evaluating individually detailed aspects of post-vaccination events and influencing the drop-off rate.

### 4.2. Implications

For public health practice, these findings confirm the safety of both types of vaccines widely used in Europe among HWs. Improvements need to be made to the current system that provides information about the safety and efficiency of the vaccination program at national level. Healthcare providers, persons and regulatory bodies are expected to act promptly, encouraging people in direct contact with patients to be fully vaccinated. While the campaign related to COVID-19 inoculation in healthcare personnel turned out to be insufficient, students were also a major concern during the initial phase of vaccination programs, at the beginning of 2021. The concept of mandatory immunisation via newly developed vaccines for front-line HWs appears viable and well justified [1]. Despite the confirmed safety profile of both types of anti-COVID-19 vaccines, further cross-comparison studies are required to convince individuals, including HWs, to increase the uptake of the vaccine. A more efficient campaign to promote COVID-19 vaccination in Poland, is urgently required, with the support of policy makers and care providers, particularly encouraging the HWs on the front line to be recipients of safe inoculation. This seems particularly valid when facing the booster vaccination program that began in September 2021 in Europe and the US.

The overall safety of COVID-19 vaccines, should provide an evidence-informed reassurance for populations so far unvaccinated, supporting global COVID-19 mitigation strategies. Long-term, vaccines surveillance data gathered from well-designed studies, with a large sample size, is warranted to ensure the safety of inoculation programs. Register of vaccinated keyworkers, including HWs, connected to electronic health databases would support national vaccine surveillance strategies. Global communication of regulatory authorities, organisations, and development of multi-national projects, such as European Accelerated Development of Vaccine Benefit-Risk Collaboration, The Vaccine Safety Datalink (US), and Vaccine COVID-19 Monitoring Readiness (ACCESS) might improve monitoring of vaccine-related adverse events via access to electronic medical records [70].

Independent targeted survey-based studies on national/local levels, as a component of pharmacovigilance programs, are essential to obtain accurate data specific for geographical regions, regarding post-vaccination SRAEs. The recruitment of participants should be promoted by medical, dental, nursing, and pharmaceutical councils, medical/dental schools, and top managers of healthcare services.

## 5. Conclusions

Polish healthcare workers and students at medical universities reported minor, local and systemic side effects mainly after both doses of the mRNA-based and the viral vector-based COVID-19 vaccinations. No major or life-threatening incidents that would affect regular functioning were reported. Primarily, young, male participants (<29 years old) experienced higher incidents of common mild systemic adverse reactions, similar to side effects associated with other vaccinations for the prevention of respiratory tract infections (influenza). With the existing limitations of this survey, its results confirmed the safety of most common COVID-19 vaccines. The international network of complementary, active vaccine safety surveillance systems could enhance the public trust and overcome vaccine hesitancy at a global level. Public health authorities should encourage and foster scientific collaboration across regions to enable data sharing and unrestricted access to information.

Widely extended cross-sectional surveys, involving separate groups of healthcare sector workers, as well as students attending medical and dental schools would enhance the public trust in national inoculation programs aimed to curb the COVID-19 pandemic. Non-vaccinated healthcare workers and medical students who deal with vulnerable and medically compromised individuals are requested to opt for fully vaccinated status in order to protect exposed persons susceptible to COVID-19 contraction and its consequences.

## Figures and Tables

**Table 1:** Demographic characteristics of the surveyed Polish healthcare workers.

Variable	Outcome	mRNA-Based (*n* = 187)	Viral Vector-Based (*n* = 60)	Total (*n* = 247)
Gender	Female	148 (79.1%)	49 (81.7%)	197 (79.8%)
	Male	39 (20.9%)	11 (18.3%)	50 (20.2%)
	Prefer not to disclose	0 (0%)	0 (0%)	0 (0%)
Age	≤29 years-old	100 (53.5%)	52 (86.7%)	152 (61.5%)
	>29 years-old	87 (46.5%)	8 (13.3%)	95 (38.5%)
Profession	Physician	30 (16%)	1 (1.7%)	31 (12.6%)
	Dentist	27 (14.4%)	7 (11.7%)	34 (13.8%)
	Nurse	9 (4.8%)	1 (1.7%)	10 (4%)
	Midwife	10 (5.3%)	0 (0%)	10 (4%)
	Laboratory Worker	12 (6.4%)	2 (3.3%)	14 (5.7%)
	Physiotherapist	2 (1.1%)	1 (1.7%)	3 (1.2%)
	Pharmacist	3 (1.6%)	0 (0%)	3 (1.2%)
	Psychologist	1 (0.5%)	1 (1.7%)	2 (0.8%)
	Student	76 (50.6%)	42 (70%)	118 (47.8%)
	Dietician	3 (1.6%)	1 (1.7%)	4 (1.6%)
	Other	14 (7.5%)	4 (6.7%)	18 (7.3%)
Region	Wielkopolskie	0 (0%)	2 (3.3%)	2 (0.8%)
	Kujawsko-Pomorskie	1 (0.5%)	0 (0%)	1 (0.4%)
	Małopolskie	10 (5.3%)	14 (23.3%)	24 (9.7%)
	Łódzkie	4 (2.1%)	0 (0%)	4 (1.6%)
	Dolnośląskie	6 (3.2%)	2 (3.3%)	8 (3.2%)
	Lubelskie	0 (0%)	1 (1.7%)	1 (0.4%)
	Lubuskie	2 (1.1%)	0 (0%)	2 (0.8%)
	Mazowieckie	3 (1.6%)	1 (1.7%)	4 (1.6%)
	Opolskie	2 (1.1%)	1 (1.7%)	3 (1.2%)
	Podlaskie	1 (0.5%)	0 (0%)	1 (0.4%)
	Pomorskie	0 (0%)	2 (3.3%)	2 (0.8%)
	Śląskie	146 (78.1%)	35 (58.3%)	181 (73.3%)
	Podkarpackie	7 (3.7%)	1(1.7%)	8 (3.2%)
	Swiętokrzystkie	3 (1.6%)	0 (0%)	3 (1.2%)
	Warmińko-mazurskie	2 (1.1%)	0 (0%)	2 (0.8%)
	Zachodnio-pomorskie	0 (0%)	1 (1.7%)	1 (0.4%)

**Table 2:** Medical history of the surveyed Polish healthcare workers.

Variable	Outcome	mRNA-Based (*n* = 187)	Viral Vector-Based (*n* = 60)	Total (*n* = 247)	*p*
Chronic Illnesses	Asthma	4 (2.1%)	0 (0%)	4 (1.6%)	0.575 *
	Blood Disease	1 (0.5%)	2 (3.3%)	3 (1.2%)	0.147 *
	Cancer	0 (0%)	0 (0%)	0 (0%)	*N/A*
	Chronic Hypertension	14 (7.5%)	0 (0%)	14 (5.7%)	0.025 *
	COPD	6 (3.2%)	0 (0%)	6 (2.4%)	0.341 *
	Diabetes Mellitus I	0 (0%)	0 (0%)	0 (0%)	*N/A*
	Diabetes Mellitus II	1 (0.5%)	0 (0%)	0 (0%)	1.000 *
	Psychological Distress	10 (5.3%)	1 (1.7%)	11 (4.5%)	0.304 *
	Rheumatoid Arthritis	2 (1.1%)	0 (0%)	2 (0.8%)	1.000 *
	Thyroid Disease	21 (11.2%)	11 (18.3%)	32 (13%)	0.154
	Other	5 (2.7%)	1 (1.7%)	6 (2.4%)	1.000 *
	Total	50 (26.7%)	14 (23.3%)	64 (25.9%)	0.600
Medical Treatments	Antibiotics	1 (0.5%)	0 (0%)	0 (0%)	1.000 *
	Anticoagulants	2 (1.1%)	1 (1.7%)	3 (1.2%)	1.000 *
	Antidepressants	11 (5.9%)	3 (5%)	14 (5.7%)	1.000 *
	Antidiabetics	3 (1.6%)	0 (0%)	3 (1.2%)	1.000 *
	Antihistamines	12 (6.4%)	4 (6.7%)	16 (6.5%)	1.000 *
	Antihypertensives	12 (6.4%)	0 (0%)	12 (4.9%)	0.043 *
	Cholesterol-lowering	4 (2.1%)	1 (1.7%)	5 (2%)	1.000 *
	Contraceptives	29 (15.5%)	14 (23.3%)	43 (17.4%)	0.164
	Corticosteroids	3 (1.6%)	0 (0%)	3 (1.2%)	1.000 *
	Thyroid Hormone	21 (11.2%)	10 (16.7%)	31 (21.6%)	0.269
	Other	15 (8%)	3 (5%)	18 (7.3%)	0.574 *
	Total	79 (42.2%)	27 (45%)	106 (42.9%)	0.708

Chi-squared test (*χ*^2^) and Fisher’s exact test (*) were used with the significance level (*p*) of <0.05. Bold values denote statistical significance.

**Table 3:** COVID-19-related anamnesis of the surveyed Polish healthcare workers.

Variable	Outcome	mRNA-Based (*n* = 187)	Viral Vector-Based (*n* = 60)	Total (*n* = 247)	*p*
Doses	One	11 (5.9%)	45 (75%)	56 (22.7%)	**<0.001**
	Two	176 (94.1%)	15 (25%)	191 (77.3%)	**<0.001**
Infection	Before 1st Dose	2 (1.1%)	3 (5%)	5 (2%)	0.094 *
	Before 2nd Dose	1 (0.5%)	0 (0%)	1 (0.4%)	1.000 *

Chi-squared test (*χ*^2^*)* and Fisher’s exact test (*) were used with the significance level (*p*) of <0.05. Bold values denote statistical significance.

**Table 4:** Local adverse effects of COVID-19 vaccines reported by Polish healthcare workers.

Variable	Outcome	mRNA-Based (*n* = 187)	Viral Vector-Based (*n* = 60)	Total (*n* = 247)	*p*
Local SE Prevalence	Pain at injection site	147 (78.6%)	43 (71.7%)	190 (76.9%)	0.267
	Swelling at injection site	32 (17.1%)	12 (20%)	44 (17.8%)	0.611
	Redness at injection site	33 (17.6%)	8 (13.3%)	41 (16.6%)	0.435
	Total	152 (81.3%)	43 (71.7%)	195 (78.9%)	0.112
Local SE Onset	Only After 1st Dose	44 (29.1%)	37 (88.1%)	81 (42%)	**<0.001**
	Only After 2nd Dose	10 (6.6%)	0 (0%)	10 (5.2%)	0.124 *
	After Both Doses	97 (64.2%)	5 (11.9%)	102 (52.8%)	**<0.001**
Local SE Duration	1 Day	61 (40.4%)	7 (16.7%)	68 (35.2%)	**0.002**
	3 Days	70 (46.4%)	22 (52.4%)	92 (47.7%)	0.915
	5 Days	14 (9.3%)	4 (9.5%)	18 (9.3%)	1.000 *
	1 Week	2 (1.3%)	7 (16.7%)	9 (4.7%)	0.001 *
	>1 Week	4 (2.6%)	2 (4.8%)	6 (3.1%)	0.635 *

Chi-squared test (*χ*^2^) and Fisher’s exact test (*) were used with the significance level (*p*) of <0.05. Bold values denote statistical significance.

**Table 5:** Systemic adverse effects of COVID-19 vaccines reported by Polish healthcare workers.

Variable	Outcome	mRNA-Based (*n* = 187)	Viral Vector-Based (*n* = 60)	Total (*n* = 247)	*p*
Systemic SE Prevalence	Fatigue	74 (39.6%)	40 (66.7%)	114 (46.2%)	**<0.001**
	Headache	58 (31%)	35 (58.3%)	93 (37.7%)	**<0.001**
	Muscle Pain	48 (25.7%)	30 (50%)	78 (31.6%)	**<0.001**
	Joint Pain	29 (15.5%)	25 (41.7%)	54 (21.9%)	**<0.001**
	Fever	41 (21.9%)	30 (50%)	71 (28.7%)	**<0.001**
	Chills	45 (24.1%)	32 (53.3%)	77 (31.2%)	**<0.001**
	Nausea	6 (3.2%)	12 (20%)	18 (7.3%)	<0.001 *
	Diarrhoea	4 (2.1%)	2 (3.3%)	6 (2.4%)	0.635 *
	Lymphadenopathy	11 (5.9%)	4 (6.7%)	15 (6.1%)	0.763 *
	Shortness of Breath	3 (1.6%)	7 (11.7%)	10 (4%)	0.002 *
	Anaphylaxis	0 (0%)	0 (0%)	0 (0%)	*N/A*
	Total	104 (55.6%)	46 (76.7%)	150 (60.7%)	**0.004**
Systemic SE Onset	Only After 1st Dose	16 (15.7%)	40 (93%)	56 (38.6%)	**<0.001**
	Only After 2nd Dose	46 (45.1%)	0 (0%)	46 (31.7%)	**<0.001**
	After Both Doses	40 (39.2%)	3 (7%)	43 (29.7%)	**<0.001**
Systemic SE Duration	1 Day	48 (47.1%)	17 (39.5%)	65 (44.8%)	0.683
	3 Days	39 (38.2%)	19 (44.2%)	58 (40%)	0.086
	5 Days	3 (2.9%)	1 (2.3%)	4 (2.8%)	1.000 *
	1 Week	4 (3.9%)	1 (2.3%)	5 (3.4%)	1.000 *
	>1 Week	3 (2.9%)	4 (9.3%)	7 (4.8%)	0.061 *
	>1 Month	5 (4.9%)	1 (2.3%)	6 (4.1%)	1.000 *

Chi-squared test (*χ*^2^) and Fisher’s exact test (*) were used with the significance level (*p*) of <0.05. Bold values denote statistical significance.

**Table 6:** Orofacial and skin-related adverse events of COVID-19 vaccines reported by Polish healthcare workers.

Variable	Outcome	mRNA-Based (*n* = 187)	Viral Vector-Based (*n* = 60)	Total (*n* = 247)	*p*
Orofacial SE Prevalence	Oral Paraesthesia	4 (2.1%)	1 (1.7%)	5 (2%)	1.000 *
	Dysgeusia	3 (1.6%)	3 (5%)	6 (2.4%)	0.156 *
	Oral Ulcers	1 (0.5%)	0 (0%)	1 (0.4%)	1.000 *
	Xerostomia	1 (0.5%)	1 (1.7%)	2 (0.8%)	0.428 *
	Facial Nerve Palsy	0 (0%)	0 (0%)	0 (0%)	*N/A*
Skin-related SE Prevalence	Skin Rash	3 (1.6%)	3 (5%)	6 (2.4%)	0.156 *
	Skin Eruptions	1 (0.5%)	2 (3.3%)	3 (1.2%)	0.147 *
	Total	10 (5.3%)	8 (13.3%)	18 (7.3%)	0.048 *

Chi-squared test (*χ*^2^) and Fisher’s exact test (*) were used with the significance level (*p*) of <0.05. Bold values denote statistical significance.

**Table 7:** Symptoms-relieving drugs after COVID-19 vaccination taken by Polish healthcare workers.

Drug	mRNA-Based (*n* = 187)	Viral Vector-Based (*n* = 60)	Total (*n* = 247)	*p*
Paracetamol	35 (18.7%)	28 (46.7%)	63 (25.5%)	**<0.001**
Pyralgin	6 (3.2%)	3 (5%)	9 (3.6%)	0.457 *
Ibuprofen	18 (9.6%)	7 (11.7%)	25 (10.1%)	0.648
Total	61 (32.6%)	34 (56.7%)	95 (38.5%)	**0.001**

Chi-squared test (*χ*^2^) and Fisher’s exact test (*) were used with the significance level (*p*) of <0.05. Bold values denote statistical significance.

**Table 8:** Local, systemic, orofacial and skin-related SRAEs and palliative drugs taken after COVID-19 vaccination stratified by gender.

Variable	Outcome	mRNA-Based Vaccine			Viral Vector-Based Vaccine			Total		
		Female (*n* = 148)	Male (*n* = 39)	*p*	Female (*n* = 49)	Male (*n* = 11)	*p*	Female (*n* = 197)	Male (*n* = 50)	*p*
Local SE Prevalence	Injection Site Pain	114 (77%)	33 (84.6%)	0.304	35 (71.4%)	8 (72.7%)	1.000 *	149 (75.6%)	41 (82%)	0.340
	Injection Site Swelling	30 (20.3%)	2 (5.1%)	**0.026**	8 (16.3%)	4 (36.4%)	0.206 *	38 (19.3%)	6 (12%)	0.229
	Injection Site Redness	27 (18.2%)	6 (15.4%)	0.677	5 (10.2%)	3 (27.3%)	0.154 *	32 (16.2%)	9 (18%)	0.766
	Total	119 (80.4%)	33 (84.6%)	0.549	35 (71.4%)	8 (72.7%)	1.000 *	154 (78.2%)	41 (82%)	0.553
Local SE Onset	Only After 1st Dose	34 (28.6%)	10 (31.3%)	0.727	29 (85.3%)	8 (100%)	0.506 *	63 (41.2%)	18 (45%)	0.589
	Only After 2nd Dose	9 (7.6%)	1 (3.1%)	0.691 *	0 (0%)	0 (0%)	*N/A*	9 (5.9%)	1 (2.5%)	0.692 *
	After Both Doses	76 (63.9%)	21 (65.6%)	0.781	5 (14.7%)	0 (0%)	0.573 *	81 (52.9%)	21 (52.5%)	0.910
Systemic SE Prevalence	Fatigue	60 (40.5%)	14 (35.9%)	0.598	34 (69.4%)	6 (54.5%)	0.481 *	94 (47.7%)	20 (40%)	0.328
	Headache	48 (32.4%)	10 (25.6%)	0.415	29 (59.2%)	6 (54.5%)	1.000 *	77 (39.1%)	16 (32%)	0.356
	Muscle Pain	39 (26.4%)	9 (23.1%)	0.677	24 (49%)	6 (54.5%)	0.739	63 (32%)	15 (30%)	0.788
	Joint Pain	23 (15.5%)	6 (15.4%)	0.981	22 (44.9%)	3 (27.3%)	0.332 *	45 (22.8%)	9 (18%)	0.459
	Fever	28 (18.9%)	13 (33.3%)	0.053	24 (49%)	6 (54.5%)	0.739	52 (26.4%)	19 (38%)	0.105
	Chills	24 (23%)	11 (28.2%)	0.496	27 (55.1%)	5 (45.5%)	0.562	61 (31%)	16 (32%)	0.888
	Nausea	4 (2.7%)	2 (5.1%)	0.606 *	11 (22.4%)	1 (9.1%)	0.435 *	15 (7.6%)	3 (6%)	1.000 *
	Diarrhoea	3 (2%)	1 (2.6%)	1.000 *	1 (2%)	1 (9.1%)	0.336 *	4 (2%)	2 (4%)	0.351 *
	Lymphadenopathy	9 (6.1%)	2 (5.1%)	1.000 *	4 (8.2%)	0 (0%)	1.000 *	13 (6.6%)	2 (4%)	0.742 *
	Shortness of Breath	3 (2%)	0 (0%)	1.000 *	6 (12.2%)	1 (9.1%)	1.000 *	9 (4.6%)	1 (2%)	0.692 *
	Anaphylaxis	0 (0%)	0 (0%)	*N/A*	0 (0%)	0 (0%)	*N/A*	0 (0%)	0 (0%)	*N/A*
	Total	80 (54.1%)	24 (61.5%)	0.403	40 (81.6%)	6 (54.5%)	0.107 *	120 (60.9%)	30 (60%)	0.906
Systemic SE Onset	Only After 1st Dose	12 (15.4%)	4 (16.7%)	0.747	35 (92.1%)	5 (100%)	0.155 *	47 (40.5%)	9 (31%)	0.377
	Only After 2nd Dose	34 (43.6%)	12 (50%)	0.315	0 (0%)	0 (0%)	*N/A*	34 (29.3%)	12 (41.4%)	0.274
	After Both Doses	32 (41%)	8 (33.3%)	0.881	3 (7.9%)	0 (0%)	1.000 *	35 (30.2%)	8 (27.6%)	0.769
Orofacial SE Prevalence	Oral Paraesthesia	4 (2.7%)	0 (0%)	0.581 *	1 (2%)	0 (0%)	1.000 *	5 (2.5%)	0 (0%)	0.586 *
	Taste Disturbance	2 (1.4%)	1 (2.6%)	0.506 *	2 (4.1%)	1 (9.1%)	0.462 *	4 (2%)	2 (4%)	0.351 *
	Oral Ulcers	1 (0.7%)	0 (0%)	1.000 *	0 (0%)	0 (0%)	*N/A*	1 (0.5%)	0 (0%)	1.000 *
	Xerostomia	1 (0.7%)	0 (0%)	1.000 *	0 (0%)	1 (9.1%)	0.183 *	1 (0.5%)	1 (2%)	0.365 *
	Facial Nerve Palsy	0 (0%)	0 (0%)	*N/A*	0 (0%)	0 (0%)	*N/A*	0 (0%)	0 (0%)	*N/A*
Skin-related SE Prevalence	Skin Rash	3 (2%)	0 (0%)	1.000 *	3 (6.1%)	0 (0%)	1.000 *	6 (3%)	0 (0%)	0.604 *
	Skin Eruptions	1 (0.7%)	0 (0%)	1.000 *	2 (4.1%)	0 (0%)	1.000 *	3 (1.5%)	0 (0%)	1.000 *
	Total	9 (6.1%)	1 (2.6%)	0.691 *	6 (12.2%)	2 (18.2%)	0.631 *	15 (7.6%)	3 (6%)	1.000 *
Palliative Drugs	Paracetamol	30 (20.3%)	5 (12.8%)	0.289	24 (49%)	4 (36.4%)	0.448	54 (27.4%)	9 (18%)	0.173
	Pyralgina	5 (3.4%)	1 (2.6%)	1.000 *	3 (6.1%)	0 (0%)	1.000 *	8 (4.1%)	1 (2%)	0.691 *
	Ibuprofen	16 (10.8%)	2 (5.1%)	0.373 *	6 (12.2%)	1 (9.1%)	1.000 *	22 (11.2%)	3 (6%)	0.279
	Total	51 (34.5%)	10 (25.6%)	0.296	29 (59.2%)	5 (45.5%)	0.507 *	80 (40.6%)	15 (30%)	0.168

Chi-squared test (*χ*^2^) and Fisher’s exact test (*) were used with the significance level (*p*) of <0.05. Bold values denote statistical significance.

**Table 9:** Local, systemic, orofacial and skin-related SRAEs and palliative drugs following COVID-19 vaccination stratified by age.

Variable	Outcome	mRNA-Based Vaccine			Viral Vector-Based Vaccine			Total		
		≤29 yo (*n* = 100)	>29 yo (*n* = 87)	*p*	≤29 yo (*n* = 52)	>29 yo (*n* = 8)	*p*	≤29 yo (*n* = 152)	>29 yo (*n* = 92)	*p*
Local SE Prevalence	Injection Site Pain	80 (80%)	67 (77%)	0.619	37 (71.2%)	6 (75%)	1.000 *	117 (77%)	73 (76.8%)	0.981
	Injection Site Swelling	18 (18%)	14 (16.1%)	0.730	9 (17.3%)	3 (37.5%)	0.191 *	27 (17.8%)	17 (17.9%)	0.979
	Injection Site Redness	15 (15%)	18 (20.7%)	0.309	5 (9.6%)	3 (37.5%)	0.065 *	20 (13.2%)	21 (22.1%)	0.066
	Total	83 (83%)	69 (79.3%)	0.519	37 (71.2%)	6 (75%)	1.000 *	120 (78.9%)	75 (78.9%)	1.000
Local SE Onset	Only After 1st Dose	20 (24.4%)	24 (34.8%)	0.223	31 (86.1%)	6 (100%)	0.698 *	51 (43.2%)	30 (40%)	0.748
	Only After 2nd Dose	3 (3.7%)	7 (10.1%)	0.192 *	0 (0%)	0 (0%)	*N/A*	3 (2.5%)	7 (9.3%)	0.048 *
	After Both Doses	59 (72%)	38 (55.1%)	**0.036**	5 (13.9%)	0 (0%)	1.000 *	64 (54.2%)	38 (50.7%)	0.744
Systemic SE Prevalence	Fatigue	41 (41%)	33 (37.9%)	0.669	36 (69.2%)	4 (50%)	0.422 *	77 (50.7%)	37 (38.9%)	0.072
	Headache	37 (37%)	21 (24.1%)	0.058	31 (59.6%)	4 (50%)	0.708 *	68 (44.7%)	25 (26.3%)	**0.004**
	Muscle Pain	29 (29%)	19 (21.8%)	0.263	28 (53.8%)	2 (25%)	0.254 *	57 (37.5%)	21 (22.1%)	**0.011**
	Joint Pain	17 (17%)	12 (13.8%)	0.546	24 (46.2%)	1 (12.5%)	0.123 *	41 (27%)	13 (13.7%)	**0.014**
	Fever	27 (27%)	14 (16.1%)	0.072	29 (55.8%)	1 (12.5%)	0.052 *	56 (36.8%)	15 (15.8%)	**<0.001**
	Chills	24 (24%)	21 (24.1%)	0.982	28 (53.8%)	4 (50%)	1.000 *	52 (34.2%)	25 (26.3%)	0.193
	Nausea	4 (4%)	2 (2.3%)	0.687 *	12 (23.1%)	0 (0%)	0.338 *	16 (10.5%)	2 (2.1%)	**0.013**
	Diarrhoea	2 (2%)	2 (2.3%)	1.000 *	2 (3.8%)	0 (0%)	1.000 *	4 (2.6%)	2 (2.1%)	1.000 *
	Lymphadenopathy	5 (5%)	6 (6.9%)	0.582	4 (7.7%)	0 (0%)	1.000 *	9 (5.9%)	6 (6.3%)	0.899
	Shortness of Breath	2 (2%)	1 (1.1%)	1.000 *	7 (13.5%)	0 (0%)	0.578 *	9 (5.9%)	1 (1.1%)	0.094 *
	Anaphylaxis	0 (0%)	0 (0%)	*N/A*	0 (0%)	0 (0%)	*N/A*	0 (0%)	0 (0%)	*N/A*
	Total	59 (59%)	45 (51.7%)	0.318	41 (78.8%)	5 (62.5%)	0.374 *	100 (65.8%)	50 (52.6%)	**0.039**
Systemic SE Onset	Only After 1st Dose	13 (22.4%)	3 (6.8%)	**0.020**	37 (94.9%)	3 (75%)	0.103 *	50 (51.5%)	6 (12.5%)	<0.001
	Only After 2nd Dose	23 (39.7%)	23 (52.3%)	0.586	0 (0%)	0 (0%)	*N/A*	23 (23.7%)	23 (47.9%)	0.075
	After Both Doses	22 (37.9%)	18 (40.9%)	0.827	2 (5.1%)	1 (25%)	0.354 *	24 (24.7%)	19 (39.6%)	0.396
Orofacial SE Prevalence	Oral Paraesthesia	2 (2%)	2 (2.3%)	1.000 *	0 (0%)	1 (12.5%)	0.133 *	2 (1.3%)	3 (3.2%)	0.376 *
	Taste Disturbance	2 (2%)	1 (1.1%)	1.000 *	3 (5.8%)	0 (0%)	1.000 *	5 (3.3%)	1 (1.1%)	0.411 *
	Oral Ulcers	0 (0%)	1 (1.1%)	0.465 *	0 (0%)	0 (0%)	*N/A*	0 (0%)	1 (1.1%)	0.385 *
	Xerostomia	1 (1%)	0 (0%)	1.000 *	1 (1.9%)	0 (0%)	1.000 *	2 (1.3%)	0 (0%)	0.525 *
	Facial Nerve Palsy	0 (0%)	0 (0%)	*N/A*	0 (0%)	0 (0%)	*N/A*	0 (0%)	0 (0%)	*N/A*
Skin-related SE Prevalence	Skin Rash	3 (3%)	0 (0%)	0.250 *	3 (5.8%)	0 (0%)	1.000 *	6 (3.9%)	0 (0%)	0.085 *
	Skin Eruptions	1 (1%)	0 (0%)	1.000 *	2 (3.8%)	0 (0%)	1.000 *	3 (2%)	0 (0%)	0.287 *
	Total	8 (8%)	2 (2.3%)	0.108 *	7 (13.5%)	1 (12.5%)	1.000 *	15 (9.9%)	3 (3.2%)	**0.048**
Palliative Drugs	Paracetamol	21 (21%)	14 (16.1%)	0.391	24 (46.2%)	4 (50%)	1.000 *	45 (29.6%)	18 (18.9%)	0.062
	Pyralgina	3 (3%)	3 (3.4%)	1.000 *	3 (5.8%)	0 (0%)	1.000 *	6 (3.9%)	3 (3.2%)	1.000 *
	Ibuprofen	9 (9%)	9 (10.3%)	0.756	7 (13.5%)	0 (0%)	0.578 *	16 (10.5%)	9 (9.5%)	0.790
	Total	34 (34%)	27 (31%)	0.666	30 (57.7%)	4 (50%)	0.717 *	64 (42.1%)	31 (32.6%)	0.137

Chi-squared test (*χ*^2^) and Fisher’s exact test (*) were used with the significance level (*p*) of <0.05. Bold values denote statistical significance.

**Table 10:** Predictors of COVID-19 vaccines SRAEs reported by Polish healthcare workers.

	mRNA-Based Vaccine		Viral Vector-Based Vaccine		Total	
Predictor	Local Side EffectsOR (CI 95%)	Systemic Side Effects OR (CI 95%)	Local Side Effects OR (CI 95%)	Systemic Side Effects OR (CI 95%)	Local Side Effects OR (CI 95%)	Systemic Side Effects OR (CI 95%)
Gender: Female	0.746 (0.286–1.948)	0.735 (0.357–1.513)	0.938 (0.217–4.055)	3.704 (0.923–14.866)	0.786 (0.354–1.744)	1.039 (0.551–1.959)
Age: ≤29 years-old	1.274 (0.610–2.658)	1.343 (0.752–2.397)	0.822 (0.149–4.542)	2.236 (0.461–10.841)	1.000 (0.533–1.875)	1.731 (1.025–2.923)
Chronic Illness: Yes	1.067 (0.461–2.468)	1.022 (0.532–1.961)	2.903 (0.575–14.654)	2.118 (0.413–10.865)	1.391 (0.666–2.906)	1.106 (0.615–1.988)
Medication: Yes	1.299 (0.609–2.769)	1.099 (0.613–1.972)	3.737 (1.049–13.319)	1.650 (0.479–5.684)	1.729 (0.908–3.292)	1.200 (0.715–2.015)
Doses: One	2.394 (0.296–19.347)	0.648 (0.191–2.203)	1.375 (0.390–4.850)	1.273 (0.332–4.875)	0.849 (0.416–1.732)	1.841 (0.964–3.515)
Palliative Drug: Yes	4.650 (1.561–13.856)	18.200 (6.811–48.636)	12.056 (2.932–49.569)	33.000 (3.911–278.471)	5.287 (2.272–12.306)	22.128 (9.105–53.779)

Binary logistic regression test was used with the significance level (*p*) of <0.05.

**Table 11:** The comparison of cross-sectional CoVaST studies results; post-vaccination adverse events in HWs associated with COVID-19 vaccine.

CoVasST Study (N)	SRAEs (Total %) Local/Systemic	mRNA Vaccine		Vector-Based Vaccine		mRNA	Vector-Based	One Dose (%) Two Doses (%)
		Local (%)	Systemic (%)	Local (%)	Systemic (%)	Female: Local/Systemic (%) Male: Local/Systemic (%)	Female: Local/Systemic (%) Male: Local/Systemic (%)	
Czech Republic (877)	91.79 82.90	91.7	82.90	-	-	90.70/82.49 95.29/74.12	- -	6.4 93.6
Slovakia (522)	85.82 70.50	85.82	70.50	-	-	88.06/75.62 78.33/53.33	- -	n/s
Germany (474)	n/s	78.27	60.97	70.40	87.20	78.22/62.75 77.87/55.74	77.38/91.67 56.10/78.05	28.4 71.6
Poland (247)	n/s	81.3	55.6	71.7	76.7	80.4/54.1 84.6/61.5	71.4/81.6 72.7/54.5	22.7 77.3

## Data Availability

Data that support the findings of this study are available from the corresponding author upon reasonable request.
